# Clinical Outcome and Costs Based on the Degree of Vitamin K Antagonist Control for Non-Valvular Atrial Fibrillation

**DOI:** 10.3390/jcm14030998

**Published:** 2025-02-04

**Authors:** M. Rosa Dalmau Llorca, Zojaina Hernández Rojas, Elisabet Castro Blanco, Noèlia Carrasco-Querol, Alessandra Queiroga Gonçalves, Anna Espuny Cid, José Fernández Sáez, Manuel García-Goñi, Julián Pérez-Villacastín, Carina Aguilar Martín

**Affiliations:** 1 Servei Atenció Primària Terres de l’Ebre, Institut Català de la Salut, 43500 Tortosa, Tarragona, Spain; zhernandezr.ebre.ics@gencat.cat (Z.H.R.); aqueiroga.ebre.ics@gencat.cat (A.Q.G.); jfernandez@idiapjgol.info (J.F.S.); 2 Unitat de Suport a la Recerca Terres de l’Ebre, Fundació Institut Universitari per a la Recerca a l’Atenció Primària de Salut Jordi Gol i Gurina, (IDIAPJGol), 43500 Tortosa, Tarragona, Spain; ecastro@idiapjgol.info (E.C.B.); ncarrasco@idiapjgol.info (N.C.-Q.); aespuny@idiapjgol.org (A.E.C.); 3 Programa de Doctorat Biomedicina, Universitat Rovira i Virgili, 43500 Tortosa, Tarragona, Spain; 4 Department of Applied & Structural Economics and History Complutense, University of Madrid, 28040 Madrid, Spain; mggoni@ccee.ucm.es; 5 Servicio de Cardiología, Hospital Clínico San Carlos, 28040 Madrid, Spain; jvillacastin@secardiologia.es; 6 Centro de Investigación Biomédica en Red de Enfermedades Cardiovasculares (CIBERCV), 28029 Madrid, Spain

**Keywords:** atrial fibrillation, acenocoumarol, warfarin, international normalized ratio, costs, cost analysis

## Abstract

**Introduction and objectives:** Adequate anticoagulation control with vitamin K antagonists (VKAs) in non-valvular atrial fibrillation (NVAF) improves health outcomes. Knowing how the economic burden depends on the degree of anticoagulation control may be relevant for decision makers. This study analyses health outcomes and costs in relation to the degree of control of anticoagulation with VKAs in NVAF in primary care using real-world data. **Methods:** The present study analyzes health outcomes and costs based on Rosendaal’s time in therapeutic range (TTR), considering values of TTR > 70% to indicate adequate control. It was carried out using data from 2018, from the perspective of the health system, with a time horizon of 1 year, in 325 Primary Care Centers in Catalonia, Spain. **Results:** A total of 42,374 real cases were analyzed, with 46.71% categorized as receiving adequate anticoagulation control. All costs were higher for poor anticoagulation control, resulting in EUR 1811.28 per patient for poor anticoagulation control compared with EUR 1609.25 per patient for adequate anticoagulation control. Adequate TTR control provided a protective effect in admissions due to cranial hemorrhage events (ORadj = 0.75; 95% CI, 0.60–0.94), gastrointestinal bleeding (ORadj = 0.66; 95% CI, 0.54–0.80), and mortality (ORadj = 0.65; 95% CI, 0.60–0.70). **Conclusions:** Adequate anticoagulation control is associated with a reduction in cranial hemorrhage event admissions, gastrointestinal bleeding admissions, and mortality. The cost arising from patients with adequate control was lower than that for patients with inadequate control. Strategies to improve anticoagulation control could improve health outcomes and costs.

## 1. Introduction

Atrial fibrillation, the most common chronic cardiac arrhythmia, affects an estimated 1.5–2.0% people in the general population, its prevalence increasing with advancing age (e.g., 16–17% in patients aged ≥80 years) [1,2,3]. It is associated with a five-fold higher risk of stroke and other thromboembolic events, and the prognosis is not different in patients with symptomatic or asymptomatic atrial fibrillation [1,4,5]. Indeed, strokes associated with atrial fibrillation are more likely to result in death or disability [4,6]. Therefore, the morbidity, mortality, and disability resulting from this condition impose a substantial economic burden on healthcare systems worldwide [1,4,7].

Approximately 85% of atrial fibrillation cases are categorized as non-valvular atrial fibrillation (NVAF) [1]. They require individualized long-term oral anticoagulation therapy, mainly involving vitamin K antagonists (VKAs) or direct oral anticoagulants (DOACs) to prevent cardioembolic complications [3,6].

In 2018, VKAs, such as acenocoumarol and warfarin, continued to be the predominant choice for anticoagulation in Spain [8]. However, the current European guideline recommends DOACs for NVAF treatment, and the recently published Spanish Therapeutic Positioning Report has also expanded its indications for DOAC use [9,10]. Nevertheless, a considerable number of patients worldwide are still treated with VKAs [11,12].

VKA therapy requires frequent international normalized ratio monitoring and dose adjustments [9]. The degree of anticoagulation control is determined by the time in therapeutic range (TTR) over the previous 6 months, calculated using the Rosendaal method [13]. Quality indicators enable the assessment of NVAF care and its outcomes, focusing on anticoagulation management during each follow-up visit [7]. Currently, the European and Spanish criteria consider values of TTR > 70% as adequate anticoagulation control [9,10].

A major concern with VKAs is the marked prevalence of poor anticoagulation control, which ranges between 39.40% and 48.26% in Spain and between 15.30% and 41.10% in European countries [12,14,15]. Poor anticoagulation control increases the risks of stroke, the main NVAF-related complication, major bleeding, and all-cause mortality [11].

Several studies in our field have evaluated the high costs of complications linked to atrial fibrillation from an economic perspective [16]. Additionally, analytical models have assessed expenses resulting from poor anticoagulation control with VKAs in NVAF patients [14]. Therefore, considering health outcomes and costs resulting from the treatment of NVAF with VKAs, according to the degree of anticoagulation control, from a health perspective and using real-world data, can yield information of relevance to decision makers.

The aim of this study is to analyze the health outcomes and costs in NVAF patients treated at Primary Care Centers in Catalonia, using real-world data, in relation to the degree of anticoagulation control achieved with VKAs.

## 2. Materials and Methods

### 2.1. Study Design and Population

This study presents a cost analysis from a healthcare system perspective, focusing on direct healthcare costs of patients with NVAF. The analysis was conducted specifically for the year 2018, using a 1-year time frame. Health outcomes were assessed using a cross-sectional study design. Using the intention-to-treat principle, the analysis compares health outcomes and costs in relation to the degree of anticoagulation control.

This article reflects part of the framework of the FANTASTIC study, which focuses exclusively on patients with NVAF attended in the 325 Primary Care Centers of the Catalan Health Institute in Catalonia.

The target population is composed of patients with NVAF treated with vitamin K antagonists (VKAs), either acenocoumarol or warfarin, and attended at Primary Care Centers in Catalonia.

The study included patients who were diagnosed with NVAF at least one year before 1 January 2018, who had received treatment with VKAs for at least 6 months in 2017, and who had undergone international normalized ratio monitoring for at least 6 months in 2017 at a Primary Care Centers in Catalonia. Patients were considered to be receiving anticoagulant treatment with VKAs if they had an active prescription 2 months before and at the beginning of the study. They were considered to have switched their anticoagulant if they had received more than 2 months of treatment with DOACs (dabigatran, rivaroxaban, apixaban, or edoxaban) in 2018. Patients diagnosed with valvular atrial fibrillation, not receiving anticoagulation treatment, receiving treatment with DOACs at study onset, or who were pregnant were excluded from the study.

The stratification variable was the quality of anticoagulation, assessed using the TTR over the previous 6 months, calculated by the Rosendaal method [13]. The TTR assessments considered INR values from both primary care and hospital settings and were restricted to patients with at least 6 INR measurements in the last 6 months. The target population was categorized into two groups according to current European and Spanish criteria: [9,10] patients with adequate anticoagulation control (TTR > 70%) and patients with poor anticoagulation control (TTR ≤ 70%).

The outcomes of the study are described below. Health-related events were classified according to the 10th edition of the International Classification of Diseases. 

Primary outcomes included the incidence of admissions due to cranial thromboembolic events (ischemic stroke, transient ischemic attack, and indeterminate stroke), cranial hemorrhagic events (intracranial hemorrhage, traumatic intracranial hemorrhage, epidural hemorrhage, subarachnoid hemorrhage, and subdural hemorrhage), gastrointestinal bleeding, hemorrhages other than those of cranial or gastrointestinal types, and all-cause mortality. The reasons for admissions related to primary outcomes were obtained from the principal diagnosis at the time of hospital admission.

Secondary outcomes included all periodic control visits (physician and nurse consultations at the Primary Care Center and use of INR monitoring strips), specialist hospital outpatient appointments, complementary tests and laboratory analyses, and the prescribed and billed anticoagulant medication (VKAs: acenocoumarol or warfarin; DOACs: dabigatran, ribaroxaban, apixaban, or edoxaban). The number of patients who switched to DOACs by the end of the study was also determined.

The covariates of this study include patient sex and age, the time elapsed since NVAF diagnosis, cardiovascular, cerebrovascular and hemorrhagic disease, morbidity, the results of the thromboembolic (CHA2DS2-VASc) [9,10] and hemorrhagic (HAS-BLED) [9,10] risk score, and whether patients were attended outside of a Primary Care Center (because they were receiving home healthcare or were institutionalized).

We used the Information System for the Development of Research in Primary Care (SIDIAP), which contains a wide range of patient data, including demographic, health, and socioeconomic information. Data collection followed the established protocols in order to guarantee patient privacy and to comply with data protection legislation [17].

**Figure 1:**
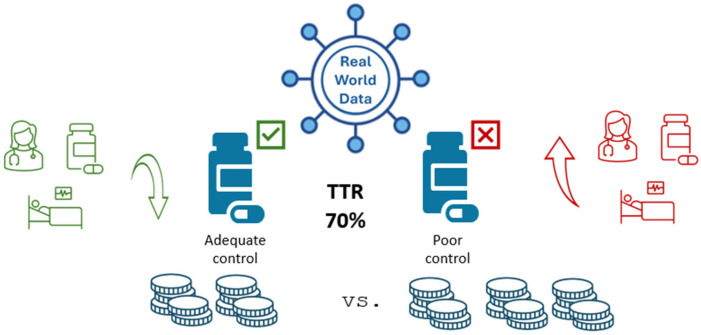
A real-world data analysis of NVAF population, health outcomes, and costs, categorized into adequate (TTR > 70%) and poor (TTR ≤ 70%) anticoagulation control. NVAF, non-valvular atrial fibrillation; TTR, time in therapeutic range.

The costs assessed included physician and nurse consultations at the Primary Care Center, INR monitoring strips, specialist hospital outpatient appointments, complementary tests related to the disease and its complications, laboratory analyses whose determinations were related to the disease, complications arising from the disease and their treatment, the invoiced cost of medications (VKAs and DOACs), and NVAF-related health events (cranial thromboembolic events, cranial hemorrhages, gastrointestinal bleeding, and all-cause mortality) (Figure 1).

The health cost analysis was based on 2018 data. All expenses were capitalized according to the 2018 Spanish annual Consumer Price Index (appraised in euros) published by the National Institute of Statistics of Spain [18]. This method was applied to expenses related to physician and nurse consultations, international normalized ratio monitoring strips, complementary tests, and analytical determinations. The costs of complementary and laboratory tests were calculated according to their categories.

NVAF-related admission expenses for outcomes, which were classified using the Diagnosis-Related Group system, were also capitalized.

Pharmacy costs were not included in the capitalization; instead, they reflect the actual invoiced costs for the Catalan Health Service in 2018, covering the real cost of the anticoagulant medication (VKAs and DOACs), including patients who switched from VKAs to DOACs (Table 1).

### 2.2. Sample Size

**Figure 2:**
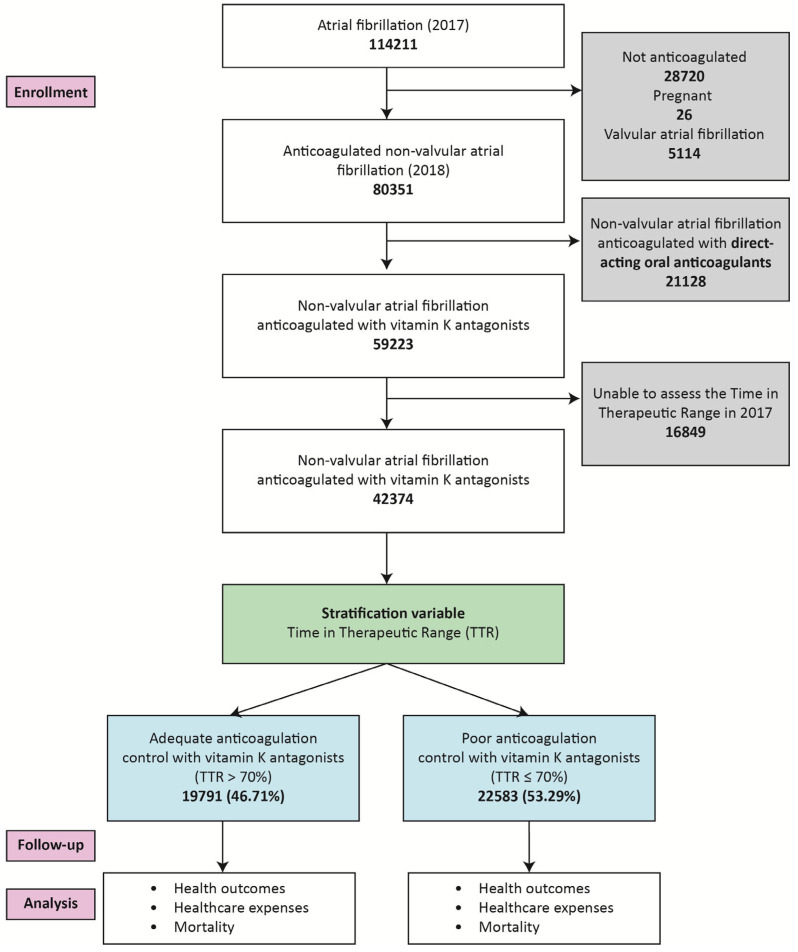
Flow diagram illustrating the selection process of the basal cohort, classification by anticoagulation control (adequate or poor), follow-up, and analysis.

The sample comprised 42,374 individuals with NVAF anticoagulated with VKAs and treated at the primary care system. Of these, 19,791 patients (46.71%) were categorized as receiving adequate anticoagulation control and 22,583 patients (53.29%) were considered to have poor anticoagulation control (Figure 2).

The statistical power of the sample was 99.27% in order to be able to conclude statistical significance for a difference of 0.003 for a rate of 2.13·100 admissions per patient-year due to cranial thromboembolic events.

### 2.3. Statistical Analysis

Statistically significant differences between the two groups were sought by performing Z tests for categorical variables and non-parametric Mann–Whitney U tests for continuous variables.

The differences in follow-up visits, admissions, and pharmacy results were evaluated using Mann–Whitney U tests. Cost differences between groups were analyzed using Hedges’ g to estimate effect sizes (small effect: g < 0.2; large effect: g > 0.8), and 95% confidence intervals (95% CI) were calculated. The cost difference and its 95% CI were also calculated.

A logistic regression was then performed to estimate the associations, as the adjusted odds ratio (ORadj), between patients admitted for cranial thromboembolic events, cranial hemorrhagic events, gastrointestinal bleeding, and all-cause mortality with other adjustment variables. Furthermore, model diagnostics and multicollinearity assessments were performed.

All analyses were carried out using R 4.2.2 and IBM SPSS Statistics v20. The threshold for statistical significance was set at 5%.

### 2.4. Sensitivity Analysis

A comprehensive sensitivity analysis capitalized all baseline population expenses from 2018 to 2024, using the variation in the Spanish annual Consumer Price Index. Subsequently, it projected three scenarios for 2025 using different discount rates: 1% for the best-case scenario, 5% for the worst-case scenario, and a rate based on the Banco de España forecast of a 2.6% increase in the Spanish annual Consumer Price Index. The analysis evaluated costs related to follow-up, admissions for health events, and pharmacy invoices to determine the total expenses of the studied population. Additionally, complementary sensitivity analyses were performed, incorporating variables like sex and diabetes mellitus.

## 3. Results

A total of 42,374 patients qualified for the study, 46.71% of whom had adequate anticoagulation control. The comparisons described below are all of the adequate control group relative to the poor control group.

In the basal cohort, the adequate control group was characterized by a higher proportion of men, younger age, and a shorter time elapsed since diagnosis. Overall, this group showed lower prior prevalences of ischemic heart disease, peripheral artery disease, ischemic and indeterminate stroke, diabetes mellitus, heart failure, kidney failure, digestive bleeding, a CHADS_2_VASC_2_ score ≥ 4 score, and a lower proportion of patients treated outside the Primary Care Center (Table 2).

Concerning follow-up visits, patients with adequate anticoagulation tended to require fewer consultations at a Primary Care Center with physicians and nurses, to use fewer INR control strips, and to undergo fewer laboratory and complementary analyses (Table 3).

In terms of admissions related to events of interest, patients with adequate anticoagulation control were less likely to experience admissions due to cranial thromboembolic events (2.13% vs. 2.46% admissions per stroke per patient per year × 100; p = 0.031), cranial hemorrhagic events (0.86% vs. 1.18% admissions per cranial hemorrhage per patient per year × 100; p = 0.006), gastrointestinal bleeding (0.90% vs. 1.49% admissions per gastrointestinal bleeding per patient per year × 100; p < 0.001), other hemorrhages (1.27% vs. 1.76% admissions per hemorrhages per patient per year × 100; p = 0.006), and all-cause mortality (5.52% vs. 9.59% deaths per patient per year × 100; p < 0.001) (Table 3).

Patients with adequate control were also more likely to continue VKA treatment (93.70% vs. 89.87%; p < 0.001) and thereby to exhibit a lower rate of switching to DOACs (6.30% vs. 10.13% patients; p < 0.001) (Table 3). 

All types of expenses appraised for the population with adequate anticoagulation control were lower: follow-up (EUR 1349.43 vs. EUR 1459.35 per patient, large effect size), admissions for health events (EUR 211.27 vs. EUR 281.32 per patient, low–moderate effect size), and pharmacy-invoiced costs (EUR 48.55 vs. EUR 70.61 per patient, moderate effect size). The total overall cost difference between the two groups was EUR 9,055,506.92 (95% CI: 9,041,681.60–9,069,332.24), with an average difference of EUR 202.03 per patient (large effect size) (Table 3).

**Figure 3:**
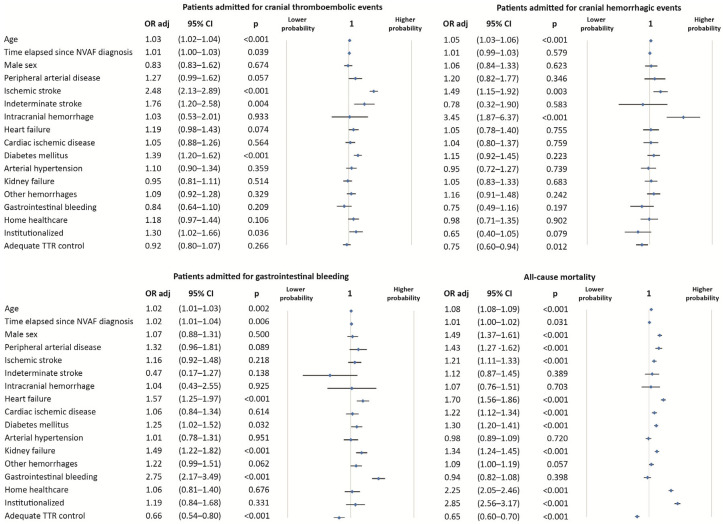
Logistic regression to study the association between patients admitted for cranial thromboembolic events, cranial hemorrhagic events, gastrointestinal bleeding and all-cause mortality with other adjustment variables, to estimate the adjusted odds ratio of adequate TTR control.

Adequate TTR control did not provide a protective effect on patients admitted for cranial thromboembolic events (OR_adj_ = 0.92; 95% CI = 0.80–1.07; p = 0.266). However, there was a protective effect on patients admitted for cranial hemorrhagic events (OR_adj_ = 0.75; 95% CI = 0.60–0.94; p = 0.012), gastrointestinal bleeding (OR_adj_ = 0.66; 95% CI = 0.54–0.80; p < 0.001), and all-cause mortality (OR_adj_ = 0.65; 95% CI = 0.60–0.70; p < 0.001) (Figure 3). Model diagnostics are available in Table S1 (Supplementary Materials). The Variance Inflation Factor was less than 5 in all the cases; therefore, multicollinearity was not present (Table S2, Supplementary Materials).

### Sensitivity Analysis

Total expenses related to NVAF patients treated with VKAs, including follow-up visits, admissions for health events, and pharmacy-invoiced costs, amounted to EUR 72,752,956.24 in 2018. When capitalized using the Spanish annual Consumer Price Index for each year until 2024, they were appraised at EUR 88,352,181.52.

For 2025, these costs were updated based on predictions for the best and worst scenarios, giving values of EUR 89,235,703.34 and EUR 92,769,790.60 for discount rates of 1% and 5%, respectively. Additionally, based on the Banco de España forecast of a 2.6% increase in the Spanish annual Consumer Price Index, the expenses amounted to EUR 90,649,338.24 (Table 4).

The sensitivity analysis stratified by sex and anticoagulation control revealed that the cost per patient for women was higher compared to the total cost per patient, regardless of whether anticoagulation control was poor or adequate (Table S3, Supplementary Materials). Similarly, the sensitivity analysis stratified by diabetes mellitus and anticoagulation control showed that the cost per patient for diabetic patients was higher compared to the total cost per patient, regardless of whether anticoagulation control was poor or adequate (Table S4, Supplementary Materials).

## 4. Discussion

The present study focused on anticoagulation control in NVAF patients, following the current criterion of a value of TTR > 70% to represent adequate anticoagulation control set out by Spain and the rest of Europe [9,10]. Adequate anticoagulation control was associated with a reduction in the likelihood of admission due to cranial hemorrhagic events and gastrointestinal bleeding and of mortality. The assessment revealed a reduction in costs for adequate anticoagulation control concerning follow-up visits, admissions for health events, and pharmacy-invoiced costs.

Using real-world clinical data, our study showed that 53.29% of the population had poor anticoagulation control (TTR ≤ 70%) in 2018. In other studies of our environment, adequate control, defined as TTR ≥ 65%, was reported in 39.40% and 57.20% of the population [14,15].

Current guidelines recommend DOACs over VKAs, and recent research supports expanding their use [9,19,20]. By the end of this study, the number of patients who switched to DOACs was determined. Specifically, patients with adequate control exhibited a lower rate of switching to DOACs and tended to remain on VKA treatment.

Costs were higher for the poor anticoagulation control group. The higher amount was attributed to follow-up visits, while expenses related to admissions for cranial thromboembolic events and cranial hemorrhagic events and treatments were lower.

The cost difference due to anticoagulation control was EUR 9.06 million, resulting in an overrun of EUR 202.03 per patient, for a total of EUR 72.75 million. Considering specifically the 22,583 patients with poor anticoagulation control, the overrun amounts to EUR 4.56 million, an expense that could be avoided by improving anticoagulation management.

In 2025, for an NVAF population of 42,374 individuals, the total cost is projected to be EUR 90.65 million. It is up to decision makers to interpret the magnitude of this expense and prioritize actions to improve anticoagulation management with VKAs.

A rise in the incidence of atrial fibrillation is anticipated over the coming decades [9]. Projections indicate that by 2030, 14–17 million patients will have this disease in the European Union and that 120,000–215,000 new cases will be diagnosed annually [3]. These estimates draw attention to the pressing need to adopt more suitable and productive measures for improving the quality of anticoagulation control [14].

When considering expenses related to NVAF across the whole of Spain, the national annual estimate for 2021 amounted to EUR 638.26 million. It was suggested that achieving adequate anticoagulation control could have saved EUR 107.90 million: EUR 79.20 million in follow-up visits and EUR 28.70 million for health events [14,16,18]. If these estimates provide a reasonable indication, the investments of the Catalan Health Service in the NVAF population treated with VKAs in 2018 could have been EUR 4.56 million less, which could have resulted in a savings of 6.27% of the total disbursement.

The sensitivity analysis also showed that costs related to follow-up and medication, including DOAC consumption, were higher in women, as evidenced in other articles [21]. In contrast, costs related to resources assigned by physicians, such as laboratory analyses, complementary tests, and admissions, were lower in women [22]. Regarding diabetes mellitus, there was an excess of costs related to the disease, similar to findings from other studies [23].

The economic data obtained represent costs of NVAF until admissions for stroke or hemorrhage. However, the data do not represent the total costs because they exclude costs incurred after admissions and omit indirect and social costs. The Spanish National Health System does not cover work incapacity and dependent care. Therefore, to understand the gap between healthcare and social perspectives, some studies estimated the costs related to incapacity and caregiving. The average annual non-healthcare expenses for strokes are EUR 18,643 per patient, driven largely by informal care (EUR 1109 million) and social care (EUR 133 million) [16,24]. Specifically for NVAF, the annual social costs for poor and adequate control were estimated at EUR 201.18 million and EUR 125.44 million, respectively [14].

The Spanish healthcare system is universal in terms of 100% medical coverage for those over 65 years of age and in terms of home care services for anticoagulation monitoring. This broad coverage ensures that patients throughout the country, regardless of their location or mobility, can receive necessary medical care, treatment, and follow-up.

Regarding health outcomes, admissions due to cranial thromboembolic events were not associated with adequate TTR control, as observed in recent European studies, which only detected an increase in hemorrhagic complications [25]. In contrast, adequate TTR control had a protective effect against cranial hemorrhagic events, digestive bleeding, and mortality.

Another factor associated with these outcomes was increased age. For cranial hemorrhagic events, previous ischemic strokes or intracranial hemorrhages increased the risk of admission. For digestive bleeding, history of digestive bleeding was the major risk factor, along with other comorbidities and disease progression time. All-cause mortality was associated with several risk factors and comorbidities, with institutionalization being the major risk. European studies evaluating thromboembolic and hemorrhagic events, using values of TTR > 70% to indicate adequate control, have also shown that poor anticoagulation control increased hemorrhagic risk but not thromboembolic or mortality risk [25].

### Strengths and Limitations

The main strength of this study is using real-world clinical data. This allowed us to gain insights into outcomes as they occurred naturally. Other strengths include the genuine reflection of medication expenses by pharmacy invoicing and the meticulous examination of all outcome and cost differences, reinforcing our confidence in the results. Additionally, we carefully considered the potential limitations. Being able to attribute observed differences with great confidence to the quality of anticoagulation control management enhances the reliability of our findings.

The main limitation lies in the assessment of the sample, which is at the population level. This can detect differences that may not be clinically relevant. However, this drawback is addressed by adjusting the logistic regression for variables that differ in the baseline cohort.

Another limitation is that mortality cannot be economically appraised from a healthcare perspective, and outcome costs were only evaluated up to the point of admission, without considering indirect and social costs.

It was not possible to obtain data on medication adherence or the socioeconomic status of the patients.

However, given the results obtained, we reaffirm the necessity of achieving adequate control to improve health outcomes in order to reduce costs.

We believe that the results of this study could be extrapolated to the entire Spanish National Health System and probably to other European regions that, like Spain, have predominantly public healthcare systems. While we believe the health results are relatively easy to extrapolate, we recognize that the economic results will be more specific to each national territory.

Given all the observations, we advocate for a more in-depth investigation in this area using real-world data to enhance the management of anticoagulation control. Future research endeavors should strive for a deeper and more comprehensive understanding of the field and explore whether adjusting the threshold for adequate anticoagulation control would contribute to a reduction in outcomes and costs. The use of innovative tools to improve anticoagulation control would be of great benefit in this field.

## 5. Conclusions

Adequate anticoagulation control was associated with a reduction in admissions for hemorrhagic cranial events, digestive bleeding, and mortality. Expenses arising from patients with adequate anticoagulation control were also lower. Implementing strategies for improving the management of anticoagulation control should improve health outcomes and reduce costs.

Therefore, we advocate for the importance of adopting more suitable strategies to enhance the quality of anticoagulation management. We emphasize the need to continue contributing to this area in future research for the purpose of effectively improving NVAF-related outcomes and reducing costs.

## Figures and Tables

**Table 1:** Source and description of variables.

Variable	Description	Source of Information	Cost Source	Temporary Adjustment
Primary outcomes				
Health-related events	Patients admitted to a hospital due to NVAF-related health outcomes, classified based on the Diagnosis-Related Group system	CMBD-AH	Official Journal of the Government of Catalonia (2012 edition)	Spanish annual Consumer Price Index update
Secondary outcomes				
Oral anticoagulant prescriptions	VKAs or DOACs prescribed	Shared electronic medical record (SIDIAP)	-	Not required
Periodic control visits at primary care	Physician or nurse consultations, including used international normalized ratio monitoring strips	Shared electronic medical record (SIDIAP)	Official Journal of the Government of Catalonia (2012 edition)	Spanish annual Consumer Price Index update
Specialist hospital outpatient appointments	Appointments for internal medicine, cardiology, neurology, hematology, and rehabilitation	Shared electronic medical record (SIDIAP)	Official Journal of the Government of Catalonia (2012 edition)	Spanish annual Consumer Price Index update
Laboratory tests	Laboratory analytical tests	Laboratory and Minimum Basic Data Set—Hospital Discharge (SIDIAP)	Official Journal of the Government of Catalonia (2012 edition)	Spanish annual Consumer Price Index update
Complementary tests	Imaging tests and other procedures	CMBD-AH	Official Journal of the Government of Catalonia (2012 edition)	Spanish annual Consumer Price Index update
Medication expenditure	Invoiced cost of anticoagulant medication for the Catalan Health Service	Pharmacy invoicing	Established public sale price—Department of Health	Not required
Covariates				
Patient characteristics	Age and sex	Shared electronic medical record (SIDIAP)	-	Not required
Morbidity	All data were collected from clinical records classified in accordance with the International Classification of Diseases (10th edition)	Shared electronic medical record and Minimum Core Hospital Discharge Data Set (SIDIAP)	-	Not required
CHA_2_DS_2_-VASC and HAD-BLED score				
Patients treated outside of a PCC	Patients receiving home healthcare or residing in institutions	Shared electronic medical record SIDIAP	-	Not required

CMBD-AH, Minimum Basic Data Set—Acute General Hospitals; DOAC, direct oral anticoagulant; NVAF, non-valvular atrial fibrillation; PCC, Primary Care Center; SIDIAP, Information System for the Development of the Primary Care Research database; VKA, vitamin K antagonist.

**Table 2:** The basal cohort, categorized into poor (TTR ≤ 70%) and adequate (TTR > 70%) anticoagulation control. Significant differences between groups are indicated in bold (p).

	Poor Anticoagulation Control	Adequate Anticoagulation Control	
	n (%)	n (%)	p
**Total Sample**	**22,583 (53.29)**	**19,791 (46.71)**	
**Patient Characteristics**			
Female	11,608 (51.40)	9623 (48.62)	**<0.001**
Male	10,975 (48.60)	10,168 (51.38)	**<0.001**
Age, Median (IQR)	81.84 (11.50)	81.25 (11.33)	<0.001 *
Time elapsed since NVAF diagnosis, Median (IQR)	6.05 (7.05)	5.95 (6.75)	0.537 *
**Cardiovascular disease**			
Peripheral arterial disease	1755 (7.77)	1229 (6.21)	**<0.001**
Ischemic heart disease	4495 (19.90)	3500 (17.68)	**<0.001**
**Cerebrovascular disease**			
Ischemic stroke	3890 (17.23)	3160 (15.97)	**0.001**
Indeterminate stroke	408 (1.81)	273 (1.38)	**<0.001**
Intracranial hemorrhage	225 (1.00)	172 (0.87)	0.175
**Morbidity**			
Diabetes mellitus	7971 (35.30)	6065 (30.65)	**<0.001**
Arterial hypertension	18,167 (80.45)	15,835 (80.01)	0.263
Heart failure	3738 (16.55)	2529 (12.78)	**<0.001**
Kidney failure	7458 (33.02)	5712 (28.86)	**<0.001**
**Hemorrhagic events**			
Non-intracranial, non-gastrointestinal hemorrhage	5732 (25.38)	4862 (24.57)	0.053
Gastrointestinal bleeding	1979 (8.76)	1508 (7.62)	**<0.001**
**CHA_2_DS_2_-VASC score**			
0	212 (0.94)	172 (0.87)	0.450
1	941 (4.17)	982 (4.96)	**<0.001**
2	3331 (14.75)	3426 (17.31)	**<0.001**
3	7590 (33.61)	7079 (35.77)	**<0.001**
≥4	10,509 (46.54)	8132 (41.09)	**<0.001**
**HAS-BLED score**			
0	241 (1.07)	172 (0.87)	**0.038**
1	3366 (14.91)	2806 (14.18)	**0.034**
2	7474 (33.10)	6478 (32.73)	0.427
3	6868 (30.41)	6241 (31.53)	**0.013**
≥4	4634 (20.52)	4094 (20.69)	0.673
**Patients attended outside of a PCC**			
Home healthcare	2924 (12.95)	1825 (9.22)	**<0.001**
Institutionalized	1549 (6.86)	855 (4.32)	**<0.001**

p * non-parametric Mann–Whitney U test; IQR, interquartile range; NVAF, non-valvular atrial fibrillation; PCC, Primary Care Center; TTR, time in therapeutic range; VKAs, vitamin K antagonists.

**Table 3:** Outcomes and costs. TTR > 70% for adequate and TTR ≤ 70% for poor anticoagulation control. All expenses in euros for 2018.

Outcomes of Interest				Costs					
	Poor Anticoagulation Control	Adequate Anticoagulation Control		Poor Anticoagulation Control	Adequate Anticoagulation Control				
**Population**	**22,583**	**19,791**		**22,583**	**19,791**				
**Periodic Control Visits**	**n (per Patient)**	**n (per Patient)**	**p**	**Total Cost** **(per Patient)**	**Total Cost** **(per Patient)**	**Total**	**Difference**	**95% CI**	**Hedges’ g (IC95%)**
Family physician consultations	286,898 (12.70)	226,792 (11.46)	<0.001	13,707,986.44 (607)	10,836,121.76 (547.53)	24,544,108.20	2,871,864.68	2,863,810.02–2,879,919.34	1.975 (1.958–1.991)
Nurse consultations	494,659 (21.90)	407,865 (20.61)	<0.001	16,536,450.37 (732.25)	13,634,926.95 (688.95)	30,171,377.32	2,901,523.42	2,892,518.50–2,910,528.34	2.437 (2.419–2.454)
Appointments for hospital outpatient specialist services	5560 (0.25)	4766 (0.24)	0.353	801,140.40 (35.48)	686,732.94 (34.70)	1,487,873.34	114,407.46	112,392.85–116,422.07	0.246 (0.232–0.26)
Used INR test strips	369,546 (16.36)	316,659 (16.00)	<0.001	162,600.24 (7.20)	139,329.96 (7.04)	301,930.20	23,270.28	22,362.81–24,177.75	3.262 (3.241–3.282)
Laboratory analyses	48,509 (2.15)	36,719 (1.86)	<0.001	1,343,751.45 (59.50)	1,045,419.21 (52.82)	2,389,170.66	298,332.24	295,827.27–300,837.21	1.173 (1.158–1.187)
Complementary tests	8231 (0.36)	7240 (0.37)	0.034	404,484.49 (17.91)	364,099.46 (18.40)	768,583.95	40,385.03	38,923.92–41,846.14	0.396 (0.383–0.41)
**Follow-up costs**				**32,956,413.39 (1459.35)**	**26,706,630.28 (1349.43)**	**59,663,043.67**	**6,249,783.11**	**6,237,162.97–6,262,403.25**	**2.623 (2.605–2.641)**
**Admissions due to health events**									
Ischemic stroke	314 (1.39)	253 (1.28)	0.344	1,659,516.24 (73.49)	1,311,180.30 (66.25)	2,970,696.54	348,335.94	345,534–351,137.88	0.129 (0.115–0.142)
Transient ischemic attack	49 (0.22)	46 (0.23)	0.995	265,565 (11.76)	235,074.30 (11.88)	500,639.30	30,490.70	29,315.08–31,666.32	0.054 (0.041–0.068)
Indeterminate stroke	192 (0.85)	122 (0.62)	0.017	740,804.98 (32.80)	439,978.89 (22.23)	1,180,783.87	300,826.09	299,167.58–302,484.60	0.1 (0.086–0.113)
Total admissions due to cranial thromboembolic events	555 (2.46)	421 (2.13)	0.031	2,665,886.22 (118.05)	1,986,233.49 (100.36)	4,652,119.71	679,652.73	676,188.22–683,117.24	0.165 (0.152–0.179)
Intracranial hemorrhage	143 (0.63)	101 (0.51)	0.126	1,060,817.04 (46.97)	737,532.62 (37.27)	1,798,349.66	323,284.42	321,162.01–325,406.83	0.08 (0.067–0.094)
Traumatic intracranial hemorrhage	10 (0.04)	8 (0.04)	0.847	49,921.88 (2.21)	31,056.05 (1.57)	80,977.93	18,865.83	18,426.68–19,304.98	0.006 (−0.007–0.02)
Epidural hemorrhage	1 (0)	0 (0)	0.349	4934.14 (0.22)	0 (0)	4934.14			−0.08 (−0.093–−0.066)
Subarachnoid hemorrhage	42 (0.19)	21 (0.11)	0.033	204,331.42 (9.05)	99,263.27 (5.02)	303,594.69	105,068.15	104,269.41–105,866.89	0.042 (0.029–0.056)
Subdural hemorrhage	70 (0.31)	41 (0.21)	0.032	328,845.88 (14.56)	193,011.92 (9.75)	521,857.80	135,833.96	134,734.55–136,933.37	0.057 (0.044–0.071)
Total admissions due to cranial hemorrhagic events	266 (1.18)	171 (0.86)	0.006	1,648,850.36 (73.01)	1,060,863.86 (53.60)	2,709,714.22	587,986.50	585,426.17–590,546.83	0.105 (0.091–0.118)
Gastrointestinal bleeding	336 (1.49)	178 (0.90)	<0.001	1,311,200.52 (58.06)	675,707.76 (34.14)	1,986,908.28	635,492.76	633,416.99–637,568.53	0.125 (0.111–0.138)
Other hemorrhages	398 (1.76)	252 (1.27)	0.006	727,217.13 (32.20)	458,460.60 (23.17)	1,185,677.73	268,756.53	267,070.87–270,442.19	0.137 (0.124–0.151)
All-cause mortality	2166 (9.59)	1093 (5.52)	<0.001						
Total hospital admissions for health events costs				6,353,154.23 (281.32)	4,181,265.71 (211.27)	10,534,419.94	2,171,888.52	2,166,814.08–2,176,962.96	0.236 (0.223–0.25)
Medications									
VKAs	20,296 (89.87)	18,544 (93.70)	<0.001	393,757 (17.44)	381,344.42 (19.27)	775,101.42	12,412.58	10,926.25–13,898.91	2.179 (2.162–2.196)
Switch to DOACs	2287 (10.13)	1247 (6.30)	<0.001	1,200,906.96 (53.18)	579,484.25 (29.28)	1,780,391.21	621,422.71	619,491.98–623,353.44	0.346 (0.332–0.359)
Total medication costs				1,594,663.96 (70.61)	960,828.67 (48.55)	2,555,492.63	633,835.29	631,386.94–636,283.64	0.509 (0.496–0.523)
Total costs				40,904,231.58 (1811.28)	31,848,724.66 (1609.25)	72,752,956.24	9,055,506.92	9,041,681.60–9,069,332.24	1.428 (1.413–1.443)

CI, confidence interval; DOACs, direct oral anticoagulants; INR, international normalized ratio; TTR, time in therapeutic range; VKAs, vitamin K antagonists. p from Mann–Whitney U test. Hedges’ g for effect size

**Table 4:** All expenses for 2018 and their subsequent capitalization to 2024 using the annual Consumer Price Index variation. An update for 2025 with predictions under three scenarios with discount rates of 1%, 2.6%, and 5% is also included.

Year	2018	2024	2025		
Consumer Price Index			1%	2.6%	5%
Population: 42,374					
Periodic Control Visits	Total Cost (EUR)	Total Cost (EUR)	Total Cost (EUR)	Total Cost (EUR)	Total Cost (EUR)
Family physician consultations	24,544,108.20	29,806,699.48	30,104,766.48	30,581,673.67	31,297,034.46
Nurse consultations	30,171,377.32	36,640,531.79	37,006,937.11	37,593,185.61	38,472,558.38
Appointments for hospital outpatient specialist services	1,487,873.34	1,806,893.66	1,824,962.60	1,853,872.90	1,897,238.34
Used INR test strips	301,930.20	366,668.15	370,334.83	376,201.52	385,001.56
Laboratory analyses	2,389,170.66	2,901,441.41	2,930,455.82	2,976,878.89	3,046,513.48
Complementary tests	768,583.95	933,378.82	942,712.61	957,646.67	980,047.76
Follow-up costs	59,663,043.67	72,455,613.31	73,180,169.44	74,339,459.26	76,078,393.98
Admissions due to health events					
Ischemic stroke	2,970,696.54	3,607,654.36	3,643,730.91	3,701,453.38	3,788,037.08
Transient ischemic attack	500,639.30	607,983.19	614,063.02	623,790.75	638,382.35
Indeterminate stroke	1,180,783.87	1,433,960.02	1,448,299.62	1,471,242.98	1,505,658.02
Intracranial hemorrhage	1,798349.66	2,183,940.33	2,205,779.74	2,240,722.78	2,293,137.35
Traumatic intracranial hemorrhage	80,977.93	98,340.70	99,324.11	100,897.56	103,257.74
Epidural hemorrhage	4934.14	5992.09	6052.01	6147.88	6291.69
Subarachnoid hemorrhage	303,594.69	368,689.53	372,376.42	378,275.46	387,124.01
Subdural hemorrhage	521,857.80	633,751.22	640,088.74	650,228.76	665,438.79
Gastrointestinal bleeding	1,986,908.28	2,412,928.49	2,437,057.78	2,475,664.63	2,533,574.92
Other hemorrhages	1,185,677.73	1,439,903.19	1,454,302.23	1,477,340.68	1,511,898.35
Total admission for health events costs	10,534,419.94	12,793,143.14	12,921,074.57	13,125,764.86	13,432,800.29
Medications					
VKAs	775,101.42	941,293.73	950,706.66	965,767.36	988,358.41
DOACs	1,780,391.21	2,162,131.35	2,183,752.66	2,218,346.76	2,270,237.92
Total medication costs	2,555,492.63	3,103,425.08	3,134,459.33	3,184,114.13	3,258,596.33
Total costs	72,752,956.24	88,352,181.52	89,235,703.34	90,649,338.24	92,769,790.60

DOACs, direct oral anticoagulants; INR, international normalized ratio; TTR, time in therapeutic range; VKAs, vitamin K antagonists.

## Data Availability

The data that support the findings of this study were obtained from the SIDIAP database (Information System for Research in Primary Care). This database is representative of the Catalan population. Restrictions apply to the availability of these data, which were used under license for this study. The authors have no authorization to share the data.

## Supplementary Materials

The following supporting information can be downloaded at: https://www.mdpi.com/article/10.3390/jcm14030998/s1. Table S1: Model diagnostics. Table S2: Multicollinearity. Table S3: Sensitivity analysis by VKA control and sex. Table S4: Sensitivity analysis by VKA control and diabetes.

## Abbreviations

Non-valvular atrial fibrillation (NVAF), vitamin K antagonist (VKA), direct oral anticoagulant (DOAC), time in therapeutic range (TTR).

## References

[1] Gómez-Doblas J.J., Muñiz J., Martin J.J.A., Rodríguez-Roca G., Lobos J.M., Awamleh P., Permanyer-Miralda G., Chorro F.J., Anguita M., Roig E. (2014). Prevalencia de fibrilación auricular en España. Resultados del estudio OFRECE. Rev. Esp. Cardiol..

[2] Cea-Calvo L., Redón J., Lozano J.V., Fernández-Pérez C., Martí-Canales J.C., Llisterri J.L., González-Esteban J., Aznar J. (2007). Prevalencia de fibrilación auricular en la población Española de 60 o más años de edad. Estudio PREV-ICTUS. Rev. Esp. Cardiol..

[3] Kirchhof P., Benussi S., Kotecha D., Ahlsson A., Atar D., Casadei B., Castellá M., Diener H.-C., Heidbuchel H., Hendriks J. (2017). Guía ESC 2016 sobre el diagnóstico y tratamiento de la fibrilación auricular, desarrollada en colaboración con la EACTS. Rev. Esp. Cardiol..

[4] Wendelboe A.M., Raskob G.E. (2016). Global Burden of Thrombosis: Epidemiologic Aspects. Circ. Res..

[5] Karakasis P., Pamporis K., Siontis K.C., Theofilis P., Samaras A., Patoulias D., Stachteas P., Karagiannidis E., Stavropoulos G., Tzikas A. (2024). Major clinical outcomes in symptomatic vs. asymptomatic atrial fibrillation: A meta-analysis. Eur. Heart J..

[6] Choi S.E., Sagris D., Hill A., Lip G.Y.H., Abdul-Rahim A.H. (2023). Atrial fibrillation and stroke. Expert Rev. Cardiovasc. Ther..

[7] Arbelo E., Aktaa S., Bollmann A., D’avila A., Drossart I., Dwight J., Hills M.T., Hindricks G., Kusumoto F.M. (2021). Quality indicators for the care and outcomes of adults with atrial fibrillation. Europace.

[8] Díaz-Guzmán J., Freixa-Pamias R., García-Alegría J., Cabeza A.-I.P., Roldán-Rabadán I., Antolin-Fontes B., Rebollo P., Llorac A., Genís-Gironés M., Escobar-Cervantes C. (2022). Epidemiology of atrial fibrillation-related ischemic stroke and its association with DOAC uptake in Spain: First national population-based study 2005 to 2018. Rev. Esp. Cardiol..

[9] Van Gelder I.C., Rienstra M., Bunting K.V., Casado-Arroyo R., Caso V., Crijns H.J.G.M., De Potter T.J.R., Dwight J., Guasti L., Hanke T. (2024). 2024 ESC Guidelines for the management of atrial fibrillation developed in collaboration with the European Association for Cardio-Thoracic Surgery (EACTS). Eur. Heart J..

[10] Ministerio de Sanidad, Agencia Española de Medicamentos y Productos Sanitarios (2024). Informe de Posicionamiento Terapéutico IPT-230/V5/08022024. Criterios y recomendaciones generales para el uso de los anticoagulantes orales directos (ACOD) en la prevención del ictus y la embolia sistémica en pacientes con fibrilación auricular no valvular (FANV)..

[11] Rivera-Caravaca J.M., Roldán V., Esteve-Pastor M.A., Valdés M., Vicente V., Marín F., Lip G.Y. (2018). Reduced Time in Therapeutic Range and Higher Mortality in Atrial Fibrillation Patients Taking Acenocoumarol. Clin. Ther..

[12] Haas S., Cate H.T., Accetta G., Angchaisuksiri P., Bassand J.-P., Camm A.J., Corbalan R., Darius H., Fitzmaurice D.A., Goldhaber S.Z. (2016). Quality of vitamin k antagonist control and 1-year outcomes in patients with atrial fibrillation: A global perspective from the GARFIELD-AF registry. PLoS ONE.

[13] Rosendaal F.R., Cannegieter S.C., Van Der Meer F.J.M., Briet E. (1993). A Method to Determine the Optimal Intensity of Oral Anticoagulant Therapy. Thromb. Haemost..

[14] Barrios V., Cinza-Sanjurjo S., Gavín O., Egocheaga I., Burgos-Pol R., Soto J., Polanco C., Suárez J., Casado M.Á. (2021). Cost and burden of poor anticoagulation control with vitamin K antagonists in patients with nonvalvular atrial fibrillation in Spain. Rev. Esp. Cardiol..

[15] Barrios V., Escobar C., Prieto L., Osorio G., Polo J., Lobos J.M., Vargas D., García N. (2015). Anticoagulation Control in Patients with Nonvalvular Atrial Fibrillation Attended at Primary Care Centers in Spain: The PAULA Study. Rev. Esp. Cardiol. Engl. Ed..

[16] Alvarez-Sabín J., Quintana M., Masjuan J., Oliva-Moreno J., Mar J., Gonzalez-Rojas N., Becerra V., Torres C., Yebenes M. (2017). Economic impact of patients admitted to stroke units in Spain. Eur. J. Health Econ..

[17] Desenvolupament de la Investigació en l’Atenció Primària (SIDIAP). https://www.sidiap.org/index.php/ca/.

[18] Instituto Nacional de Estadística. https://www.ine.es/calcula/.

[19] National Institute for Health and Care Excellence, Royal College of Physicians (2021). Atrial Fibrillation: Diagnosis and Management NICE Guideline.

[20] Lavalle C., Pierucci N., Mariani M.V., Piro A., Borrelli A., Grimaldi M., Rossillo A., Notarstefano P., Compagnucci P., Russo A.D. (2024). Italian Registry in the Setting of Atrial Fibrillation Ablation with Rivaroxaban-IRIS. Minerva Cardiol. Angiol..

[21] Llorca M.R.D., Martín C.A., Carrasco-Querol N., Rojas Z.H., Drago E.F., Cumplido D.R., Blanco E.C., Vilaubí J.M.P., Gonçalves A.Q., Fernández-Sáez J. (2021). Gender and socioeconomic inequality in the prescription of direct oral anticoagulants in patients with non-valvular atrial fibrillation in primary care in catalonia (Fantas-TIC study). Int. J. Environ. Res. Public Health.

[22] Kumar N., Echouffo-Tcheugui J.B. (2021). Diabetes and atrial fibrillation in hospitalized patients in the United States. Clin. Cardiol..

[23] Park J., Bigman E., Zhang P. (2022). Productivity Loss and Medical Costs Associated with Type 2 Diabetes Among Employees Aged 18–64 Years with Large Employer-Sponsored Insurance. Diabetes Care.

[24] Luengo-Fernandez R., Violato M., Candio P., Leal J. (2020). Economic burden of stroke across Europe: A population-based cost analysis. Eur. Stroke J..

[25] Zulkifly H., Lip G.Y.H., Lane D.A. (2022). Anticoagulation Control in Older Atrial Fibrillation Patients Receiving Vitamin K Antagonist Therapy for Stroke Prevention. Int. J. Clin. Pract..

