# Supplementary material for: Clinical Outcome and Costs Based on the Degree of Vitamin K Antagonist Control for Non-Valvular Atrial Fibrillation
Source: J Clin Med. 2025 Feb 4;14(3):998. doi: 10.3390/jcm14030998 (PMC11818192; doi:10.3390/jcm14030998)
Supplement: Supplementary file 1 [file jcm-14-00998-s001.zip › jcm-3442389-supplementary.pdf]

Supplementary material

Table S1: Model diagnostics

Model	AIC	BIC	Log-likelihood	Residual deviance
Cranial thromboembolic events	7,391	7,547	-3,678	7,355
Cranial hemorrhagic events	3,830	3,986	3,795	-1,897
Gastrointestinal bleeding	4,632	4,788	4,596	-2,298
All-cause mortality	19,829	19,985	19,793	-9,897

AIC: Akaike Information Criterion. BIC: Bayesian Information Criterion.

Table S2: Multicollinearity

	Variance Inflaction Factor			
	Cranial thromboembolic events	Cranial hemorrhagic events	Gastrointestinal bleeding	All-cause mortality
Age	1.21	1.18	1.21	1.18
Time elapsed since NVAF diagnosis	1.02	1.02	1.02	1.01
Male Sex	1.10	1.09	1.11	1.13
Peripheral arterial disease	1.04	1.04	1.04	1.04
Ischemic stroke	1.05	1.03	1.03	1.03
Indeterminate stroke	1.02	1.01	1.01	1.01
Intracranial hemorrhage	1.00	1.01	1.00	1.00
Heart failure	1.07	1.06	1.08	1.05
Cardiac ischemic disease	1.04	1.04	1.04	1.04
Diabetes mellitus	1.05	1.05	1.06	1.05
Arterial hypertension	1.04	1.04	1.05	1.04
Kidney failure	1.07	1.07	1.09	1.06
Other hemorrahages	1.02	1.02	1.02	1.02
Gastrointestinal bleeding	1.01	1.01	1.01	1.01
Home healthcare	1.13	1.12	1.13	1.12
Institutionalized	1.08	1.04	1.07	1.07
Adequate TTR control	1.01	1.01	1.01	1.01

Table S3: Sensitivity analysis by VKA control and sex

	Poorly controlled				Adequate control			
	**Women**	**Men**			**Women**	**Men**		
	**11608**	**10975**			**9623**	**10168**		
	**Total cost (per patient)**	**Total cost (per patient)**	**Difference (95% CI)**	**Hedges' g (95% CI)**	**Total**	**Total cost (per patient)**	**Difference (IC 95%)**	**g hedges (IC 95%)**
**Periodic control visits**								
Physician consultations at PPC	7306708.72 (629.45)	6401277.72 (583.26)	905431 (899291.1 - 911570.9)	1.985 (1.962 - 2.007)	5536268.6 (575.32)	5299853.16 (521.23)	236415.4 (230869.1 - 241961.8)	1.985 (1.962 - 2.007)
Nurse consultations at PPC	8548117.86 (736.4)	7988332.51 (727.87)	559785.4 (552962.5 - 566608.2)	2.287 (2.263 - 2.311)	6734339.78 (699.82)	6900587.17 (678.66)	-166247.4 (-172540.4 - -159954.4)	2.287 (2.263 - 2.311)
Specialist physicians consultations	445094.01 (38.34)	356046.39 (32.44)	89047.6 (87589 - 90506.3)	0.25 (0.232 - 0.269)	383423.49 (39.84)	303309.45 (29.83)	80114 (78766.5 - 81461.5)	0.25 (0.232 - 0.269)
Used INR test strips	84398.6 (7.27)	78201.64 (7.13)	6197 (5521.4 - 6872.5)	3.463 (3.434 - 3.492)	67951.84 (7.06)	71378.12 (7.02)	-3426.3 (-4065 - -2787.6)	3.463 (3.434 - 3.492)
Laboratory analyses	657531.9 (56.64)	686219.55 (62.53)	-28687.7 (-30669.1 - -26706.2)	1.142 (1.122 - 1.162)	489294.68 (50.85)	556124.53 (54.69)	-66829.9 (-68600.8 - -65058.9)	1.142 (1.122 - 1.162)
Complentary tests	187734.04 (16.17)	216750.45 (19.75)	-29016.4 (-30120.6 - -27912.3)	0.432 (0.413 - 0.451)	178517.85 (18.55)	185581.62 (18.25)	-7063.8 (-8094.5 - -6033)	0.432 (0.413 - 0.451)
**Follow up expenses**	**17229585.13 (1484.29)**	**15726828.26 (1432.97)**	**1502756.9 (1493165.1 - 1512348.6)**	**2.541 (2.516 - 2.566)**	**13389796.24 (1391.44)**	**13316834.05 (1309.68)**	**72962.2 (64198.2 - 81726.1)**	**2.541 (2.516 - 2.566)**
**Admissions due to health events**								
Ischaemic stroke	959720.23 (82.68)	699796.01 (63.76)	259924.2 (257864.4 - 261984.1)	0.133 (0.114 - 0.151)	754467.56 (78.4)	556712.74 (54.75)	197754.8 (195919.3 - 199590.4)	0.133 (0.114 - 0.151)
Transient ischaemic attack	122619 (10.56)	142946.2 (13.02)	-20327.2 (-21223.1 - -19431.3)	0.06 (0.042 - 0.079)	100980.3 (10.49)	134094 (13.19)	-33113.7 (-33971.8 - -32255.6)	0.06 (0.042 - 0.079)
Indeterminate stroke	366965.15 (31.61)	373839.83 (34.06)	-6874.7 (-8340.1 - -5409.2)	0.109 (0.091 - 0.128)	211218.32 (21.95)	228760.57 (22.5)	-17542.3 (-18682.7 - -16401.8)	0.109 (0.091 - 0.128)

**Admissions due to craneal thromboembolic events**	1449304.38 (124.85)	1216582.04 (110.85)	232722.3 (230036.4 - 235408.3)	0.174 (0.155 - 0.192)	1066666.18 (110.85)	919567.31 (90.44)	147098.9 (144768.7 - 149429)	0.174 (0.155 - 0.192)
Intracranial haemorrhage	512472 (44.15)	548345.04 (49.96)	-35873 (-37640.6 - -34105.5)	0.084 (0.065 - 0.102)	299369.06 (31.11)	438163.56 (43.09)	-138794.5 (-140332.8 - -137256.2)	0.084 (0.065 - 0.102)
Traumatic intracranial haemorrhage	33378 (2.88)	16543.88 (1.51)	16834.1 (16508.5 - 17159.8)	0.023 (0.004 - 0.041)	22638.99 (2.35)	8417.06 (0.83)	14221.9 (13985.5 - 14458.4)	0.023 (0.004 - 0.041)
Epidural haemorrhage	4934.14 (0.43)	0 (0)	4934.1 (4934.1 - 4934.1)	-0.012 (-0.03 - 0.007)	0 (0)	0 (0)	0 (0 - 0)	-0.012 (-0.03 - 0.007)
Subarachnoid haemorrhage	137575.41 (11.85)	66756.01 (6.08)	70819.4 (70164.3 - 71474.5)	0.057 (0.039 - 0.076)	52534.07 (5.46)	46729.2 (4.6)	5804.9 (5280.9 - 6328.8)	0.057 (0.039 - 0.076)
Subdural haemorrhage	143380.29 (12.35)	185465.59 (16.9)	-42085.3 (-43096.8 - -41073.8)	0.07 (0.051 - 0.088)	83880.37 (8.72)	109131.55 (10.73)	-25251.2 (-26026.7 - -24475.7)	0.07 (0.051 - 0.088)
**Admissions due to craneal haemorrhagic events**	831739.84 (71.65)	817110.52 (74.45)	14629.3 (12456.2 - 16802.4)	0.111 (0.093 - 0.13)	458422.49 (47.64)	602441.37 (59.25)	-144018.9 (-145839.4 - -142198.3)	0.111 (0.093 - 0.13)
Gastrointestinal bleeding	665654.05 (57.34)	645546.47 (58.82)	20107.6 (18174 - 22041.2)	0.143 (0.125 - 0.162)	341594.83 (35.5)	334112.93 (32.86)	7481.9 (6091.8 - 8872)	0.143 (0.125 - 0.162)
Other haemorrhages	259872.99 (22.39)	467344.14 (42.58)	-207471.2 (-209032.2 - -205910.1)	0.149 (0.13 - 0.167)	119534.79 (12.42)	338925.81 (33.33)	-219391 (-220672.2 - -218109.8)	0.149 (0.13 - 0.167)
**Total hospital admissions by outcome of interest expenses**	**3206571.26 (276.24)**	**3146583.17 (286.7)**	**59988.1 (55723.2 - 64252.9)**	**0.253 (0.234 - 0.271)**	**1986218.29 (206.4)**	**2195047.42 (215.88)**	**-208829.1 (-212355.9 - -205302.4)**	**0.253 (0.234 - 0.271)**
**Medications**								
VKAS	201516.4 (17.36)	192240.6 (17.52)	9275.8 (8219.2 - 10332.4)	2.14 (2.117 - 2.163)	183510.97 (19.07)	197833.45 (19.46)	-14322.5 (-15383.5 - -13261.5)	2.14 (2.117 - 2.163)
Switch to DOACs	679038.42 (58.5)	521868.54 (47.55)	157169.9 (155398.3 - 158941.4)	0.397 (0.378 - 0.415)	312900.33 (32.52)	266583.92 (26.22)	46316.4 (45060.6 - 47572.2)	0.397 (0.378 - 0.415)
**Total medication expenses**	**880554.82 (75.86)**	**714109.14 (65.07)**	**166445.7 (164382.2 - 168509.2)**	**0.536 (0.517 - 0.555)**	**496411.3 (51.59)**	**464417.37 (45.67)**	**31993.9 (30349 - 33638.9)**	**0.536 (0.517 - 0.555)**

**Total expenditures**	**21316711.21 (1836.38)**	**19587520.57 (1784.74)**	**1729190.6 (1718492 - 1739889.3)**	**1.42 (1.399 - 1.44)**	**15872425.83 (1649.43)**	**15976298.84 (1571.23)**	**-103873 (-113462.7 - -94283.3)**	**1.42 (1.399 - 1.44)**

Table S4: Sensitivity analysis by VKA control and diabetes

	Poorly controlled				Adequate control			
	Non-diabetics	Diabetics			Non-diabetics	Diabetics		
	11608	10975			9623	10168		
	Total cost (per patient)	Total cost (per patient)	Difference (95% CI)	Hedges' g (95% CI)	Total	Total cost (per patient)	Difference (IC 95%)	g hedges (IC 95%)
**Periodic control visits**								
Physician consultations at PPC	8571206.42 (586.59)	5136780.02 (644.43)	3434426.4 (3428763.2 - 3440089.6)	1.985 (1.963 - 2.008)	7269296.98 (529.6)	3566824.78 (588.1)	3702472.2 (3697687.4 - 3707257)	1.987 (1.963 - 2.011)
Nurse consultations at PPC	10266219.28 (702.59)	6270231.09 (786.63)	3995988.2 (3989739.8 - 4002236.5)	2.288 (2.264 - 2.311)	9170317.02 (668.1)	4464609.93 (736.13)	4705707.1 (4700351.1 - 4711063.1)	2.705 (2.678 - 2.733)
Specialist physicians consultations	528233.94 (36.15)	272906.46 (34.24)	255327.5 (254008.5 - 256646.4)	0.251 (0.233 - 0.27)	460799.82 (33.57)	225933.12 (37.25)	234866.7 (233662.4 - 236071)	0.258 (0.238 - 0.278)
Used INR test strips	104824.72 (7.17)	57775.52 (7.25)	47049.2 (46445 - 47653.4)	3.537 (3.507 - 3.566)	96392.12 (7.02)	42937.84 (7.08)	53454.3 (52926 - 53982.5)	4.614 (4.576 - 4.652)
Laboratory analyses	779550.81 (53.35)	564200.64 (70.78)	215350.2 (213499.5 - 217200.8)	1.145 (1.125 - 1.165)	664087.44 (48.38)	381331.77 (62.87)	282755.7 (281207.9 - 284303.4)	1.298 (1.276 - 1.319)
Complentary tests	253165.58 (17.33)	151318.91 (18.98)	101846.7 (100874.5 - 102818.9)	0.435 (0.417 - 0.454)	255580.7 (18.62)	108518.77 (17.89)	147061.9 (146219.6 - 147904.3)	0.412 (0.392 - 0.431)
**Follow up expenses**	**20503200.75 (1403.18)**	**12453212.64 (1562.31)**	**8049988.1 (8041178.8 - 8058797.4)**	**2.541 (2.516 - 2.566)**	**17916474.08 (1305.29)**	**8790156.21 (1449.33)**	**9126317.9 (9118806.4 - 9133829.3)**	**2.775 (2.747 - 2.803)**
**Admissions due to health events**								
Ischaemic stroke	1056254.59 (72.29)	603261.65 (75.68)	452992.9 (451045.5 - 454940.4)	0.133 (0.115 - 0.152)	783521.62 (57.08)	527658.68 (87)	255862.9 (254063.4 - 257662.5)	0.128 (0.109 - 0.148)
Transient ischaemic attack	164257 (11.24)	101308.2 (12.71)	62948.8 (62155.1 - 63742.5)	0.061 (0.043 - 0.08)	148847.6 (10.84)	86226.71 (14.22)	62620.9 (61885.4 - 63356.4)	0.059 (0.04 - 0.079)
Indeterminate stroke	396360.3 (27.13)	344444.68 (43.21)	51915.6 (50490.4 - 53340.8)	0.11 (0.091 - 0.128)	209084.8 (15.23)	230894.09 (38.07)	-21809.3 (-22953.2 - -20665.4)	0.098 (0.078 - 0.117)
**Admissions due to craneal thromboembolic events**	1616871.89 (110.65)	1049014.53 (131.6)	567857.4 (565312.9 - 570401.8)	0.174 (0.155 - 0.192)	1141454.02 (83.16)	844779.48 (139.29)	296674.5 (294413.9 - 298935.1)	0.159 (0.139 - 0.178)
Intracranial haemorrhage	641017.06 (43.87)	419799.98 (52.67)	221217.1 (219608.6 - 222825.6)	0.084 (0.066 - 0.103)	532116.76 (38.77)	205415.86 (33.87)	326700.9 (325535.4 - 327866.4)	0.081 (0.062 - 0.101)

Traumatic intracranial haemorrhage	34248.73 (2.34)	15673.15 (1.97)	18575.6 (18257 - 18894.2)	0.025 (0.006 - 0.043)	31056.05 (2.26)	0 (0)	31056.1 (31056.1 - 31056.1)	0.021 (0.001 - 0.041)
Epidural haemorrhage	4934.14 (0.34)	0 (0)	4934.1 (0 - 0)	-0.006 (-0.024 - 0.013)	0 (0)	0 (0)	0 (0 - 0)	-0.94 (-0.961 - -0.919)
Subarachnoid haemorrhage	123063.24 (8.42)	81268.18 (10.2)	41795.1 (41087.8 - 42502.3)	0.058 (0.04 - 0.076)	68497.46 (4.99)	30765.81 (5.07)	37731.7 (37284.7 - 38178.6)	0.042 (0.022 - 0.061)
Subdural haemorrhage	222036.27 (15.2)	106809.61 (13.4)	115226.7 (114397.6 - 116055.7)	0.07 (0.052 - 0.089)	135833.95 (9.9)	57177.97 (9.43)	78656 (78044.2 - 79267.7)	0.055 (0.035 - 0.074)
**Admissions due to craneal haemorrhagic events**	1025299.44 (70.17)	623550.92 (78.23)	401748.5 (399777.5 - 403719.6)	0.112 (0.093 - 0.13)	767504.22 (55.92)	293359.64 (48.37)	474144.6 (472750.9 - 475538.2)	0.101 (0.081 - 0.121)
Gastrointestinal bleeding	777180.91 (53.19)	534019.61 (67)	243161.3 (241353.7 - 244968.9)	0.144 (0.125 - 0.162)	436988.85 (31.84)	238718.91 (39.36)	198269.9 (197041.1 - 199498.8)	0.108 (0.088 - 0.128)
Other haemorrhages	432259.53 (29.58)	294957.6 (37)	137301.9 (135957.8 - 138646)	0.149 (0.131 - 0.168)	269093.97 (19.6)	189366.63 (31.22)	79727.3 (78652.9 - 80801.8)	0.137 (0.117 - 0.157)
**Total hospital admissions by outcome of interest expenses**	**3851611.77 (263.59)**	**2501542.66 (313.83)**	**1350069.1 (1346140.2 - 1353998)**	**0.253 (0.235 - 0.272)**	**2615041.06 (190.52)**	**1566224.66 (258.24)**	**1048816.4 (1045689.1 - 1051943.7)**	**0.218 (0.198 - 0.238)**
**Medications**								
VKAS	247499.71 (16.94)	146257.29 (18.35)	101242.4 (100285.9 - 102199)	2.157 (2.134 - 2.18)	260041.32 (18.95)	121303.1 (20)	138738.2 (137852.9 - 139623.5)	2.553 (2.526 - 2.579)
Switch to DOACs	746926.99 (51.12)	453979.97 (56.95)	292947 (291265.1 - 294628.9)	0.398 (0.379 - 0.416)	381664.73 (27.81)	197819.52 (32.62)	183845.2 (182722.5 - 184967.9)	0.302 (0.282 - 0.322)
**Total medication expenses**	**994426.7 (68.06)**	**600237.26 (75.3)**	**394189.4 (392254.5 - 396124.3)**	**0.537 (0.518 - 0.556)**	**641706.05 (46.75)**	**319122.62 (52.62)**	**322583.4 (321153.5 - 324013.4)**	**0.511 (0.491 - 0.531)**
**Total expenditures**	**25349239.22 (1734.82)**	**15554992.56 (1951.45)**	**9794246.7 (9784408.6 - 9804084.8)**	**1.42 (1.399 - 1.44)**	**21173221.19 (1542.56)**	**10675503.49 (1760.18)**	**10497717.7 (10489454.8 - 10505980.6)**	**1.456 (1.434 - 1.478)**
